# A high ratio of G1 to G0 phase cells and an accumulation of G1 phase cells before S phase progression after injurious stimuli in the proximal tubule

**DOI:** 10.14814/phy2.12173

**Published:** 2014-10-07

**Authors:** Takamasa Iwakura, Yoshihide Fujigaki, Tomoyuki Fujikura, Naro Ohashi, Akihiko Kato, Hideo Yasuda

**Affiliations:** 1 Internal Medicine I, Division of Nephrology, Hamamatsu University School of Medicine, Hamamatsu, Japan; 2 Department of Internal Medicine, Teikyo University School of Medicine, Tokyo, Japan; 3 Blood Purification Unit, Hamamatsu University School of Medicine, Hamamatsu, Japan

**Keywords:** Cell cycle, G0‐G1 transition, G1 arrest, proximal tubule

## Abstract

Proximal tubule (PT) cells can proliferate explosively after injurious stimuli. To investigate this proliferative capacity, we examined cell cycle status and the expression of cyclin‐dependent kinase inhibitor p27, a G1 phase mediator, in PT cells after a proliferative or injurious stimulus. Rats were treated with lead acetate (proliferative stimulus) or uranyl acetate (UA; injurious stimulus). Isolated tubular cells were separated into PT and distal tubule (DT) cells by density‐gradient centrifugation. Cell cycle status was analyzed with flow cytometry by using the Hoechst 33342/pyronin Y method. Most PT and DT cells from control rats were in G0/G1 phase, with a higher percentage of PT cells than DT cells in G1 phase. Lead acetate and UA administration promoted the G0‐G1 transition and the accumulation of G1 phase cells before S phase progression. In PT cells from rats treated with lead acetate or a subnephrotoxic dose of UA, p27 levels increased or did not change, possibly reflecting G1 arrest. In contrast, p27 became undetectable before the appearance of apoptotic cells in rats treated with a nephrotoxic dose of UA. The decrease in p27 might facilitate rapid cell cycling. The decreased number of p27‐positive cells was associated with PT cell proliferation in renal tissues after a proliferative or injurious stimulus. The findings suggest that a high ratio of G1 to G0 phase cells and a rapid accumulation of G1 phase cells before S phase progression in the PT is a biological strategy for safe, timely, and explosive cell proliferation in response to injurious stimuli.

## Abstract (precis)

Our results suggest that the ratio of G1 to G0 phase cells in the proximal tubule is higher than that in the distal tubule under physiological conditions. They also indicate that G1 phase cells accumulate rapidly before S phase progression in response to a proliferative or injurious stimulus. Our findings implicate p27 in G1 phase cell accumulation and S phase progression in the proximal tubule.

## Introduction

Renal tubules maintain normal function and architecture through dynamic, complementary processes that balance the rate of cell elimination and the rate of cell proliferation. According to the mitosis index or proliferation index, proximal tubule (PT) cells have a low turnover rate; most are quiescent under physiological conditions (McCreight and Sulkin 1959; Litvak and Baserga 1964; Toback et al. 1993). Because normal PT cells are immunohistochemically negative for Ki67, a marker of G1 phase, most PT cells were thought to be in G0 phase (Nadasdy et al. 1994; Hall et al. 2008). However, it has been reported that Ki67 expression is negative in early G1 phase as well as in G0 phase (Gerdes et al. 1984).

PT cells can actively proliferate after an acute tubular injury to repair the PT. A genetic fate mapping study demonstrated that, after an acute tubular injury, most proliferative cells derived from intrinsic tubular cells (Humphreys et al. 2008). Vogetseder et al. (2008) have proposed that PT cells are not quiescent, but resting in G1 phase of the cell cycle under physiological conditions, thus allowing them to divide rapidly in response to certain stimuli. This proposal was based on the finding that 40% of cells in the S3 segment of the PT and 20% of cells in the S1 and S2 segments of the PT were positive for cyclin D1, a mid to late G1 phase marker, under physiological condition in rats (Vogetseder et al. 2008), indicating that many PT cells are in G1 phase, probably because of G1 arrest. The G1 arrest of PT cells was also supported by the finding that many PT cells were immunohistochemically positive for p27 (Vogetseder et al. 2008), a cyclin dependent kinase inhibitor and G1 phase mediator (Sutterlüty et al. 1999; Kamura et al. 2004).

Cells can be separated according to cell cycle status (G0/G1, S, and G2/M) by using flow cytometry to analyze the DNA content of isolated cells. In addition, G1 and G0 phase cells can be separated based on their RNA content by using the Hoechst/pyronin Y method (Shapiro 1981). Cdt1, an essential protein for chromosome replication, is specifically expressed in G1 phase cells (Wohlschlegel et al. 2000; Nishitani et al. 2001; Xouri et al. 2004, 2007; Sakaue‐Sawano et al. 2008). Cdt1 is broken down rapidly at S phase progression and in response to DNA damage (Hall et al. 1988; Higa et al. 2003; Ralph et al. 2006; Cook 2009). It is also broken down at the induction of G0 phase in response to serum deprivation (Xouri et al. 2004). In addition, p27 proteolysis in the nucleus has been associated with the G0‐G1 transition (Kamura et al. 2004) and the G1‐S transition (Sutterlüty et al. 1999).

In the present study, we investigated the relationship between cell cycle status and the rapid and explosive proliferative capacity of PT cells after injurious stimuli in rats. In isolated PT and distal tubule (DT) cells, we examined the cell cycle status, especially the ratio of G1 to G0 phase cells under physiological conditions, and cell cycle transitions in response to a proliferative stimulus or an injurious stimulus that induces reversible PT cell injury, using flow cytometry or immunocytochemistry of Cdt1. The expression of p27 in PT cells at the time of G0‐G1 and G1‐S transitions was assessed. Our results suggest that the ratio of G1 to G0 phase cells in the PT is higher than that in the DT under physiological conditions. They also indicate that G1 phase cells accumulate rapidly before an explosive S phase progression in response to a proliferative or injurious stimulus. Our findings implicate p27 in G1 phase cell accumulation and S phase progression in the PT.

## Materials and Methods

### Rats

Male Sprague‐Dawley rats weighing 230–300 g (SLC Co., Shizuoka, Japan) were provided standard rat chow and drinking water ad libitum. The experimental protocol was approved by the Ethics Review Committee for Animal Experimentation of Hamamatsu University School of Medicine.

### Reagents

Lead acetate was purchased from Wako Pure Chemical Industries, Ltd. (Osaka, Japan). Uranyl acetate (UA) dihydrate (purity >98.0%) was purchased from Fluka (Buchs, Switzerland). Collagenase type II was from Worthington Biochemical Corp. (Lakewood, NJ). Percoll was purchased from GE Healthcare UK Ltd. (Little Chalfont, Buckinghamshire, UK). Trypan blue solution, propidium iodide, Hoechst 33342, pyronin Y, and 4′,6‐diamidino‐2‐phenylindole (DAPI) were purchased from Sigma‐Aldrich Co. (St. Louis, MO). Hank's balanced salt solution (HBSS) was from Invitrogen (Carlsbad, CA). Can Get Signal^®^ solution B was from Toyobo Life Science Department (Osaka, Japan). Citrate buffer solution was from Mitsubishi Chemical Medience (Tokyo, Japan). Histofine SAB‐PO kit was from Nichirei Bioscience (Tokyo, Japan). Vector Red Alkaline Phosphatase Substrate Kit I was from Vector Laboratories (Burlingame, CA). ApopTag Plus In Situ Apoptosis Detection Kit was from Chemicon‐Millipore (Temecula, CA). The following antibodies were used: anti‐Cdt1 (rabbit polyclonal IgG, #sc‐28262; Santa Cruz Biotechnology, Santa Cruz, CA), anti‐megalin (goat polyclonal IgG, #sc‐16478; Santa Cruz Biotechnology), anti‐Ki67 (rabbit monoclonal IgG, #RM‐9106‐S; Thermo Fisher Scientific, Runcorn, Cheshire, UK), anti‐vimentin (mouse monoclonal IgG, #V6630; Sigma‐Aldrich Co.), anti‐p27 (rabbit polyclonal IgG, #ab7961; Abcam plc, Cambridge, UK), Alexa Fluor 633‐conjugated donkey anti‐goat IgG (Invitrogen), Alexa Fluor 546‐conjugated goat anti‐rabbit IgG (Invitrogen), Histofine Simple Stain Max PO (Nichirei Bioscience), Histofine Simple Stain AP (Nichirei Bioscience), GAPDH (mouse monoclonal IgG, #sc‐32233; Santa Cruz Biotechnology). Protease Inhibitor Complete Mini^®^ and the phosphatase inhibitor PhosSTOP^®^ were from Roche (Mannheim, Germany). ImmunoPure Lane Marker Reducing Sample Buffer^®^ was from Thermo Fisher Scientific (Waltham, MA).

### Experimental protocol

In total, 132 rats were used in the present study. The rats were divided into three groups. The first group (*N* = 36) received 38 mg/kg of lead acetate intravenously (Vogetseder et al. 2007), which induces the proliferation of tubular cells without inducing tubular necrosis (Choie and Richter 1974), via activation of the mitogen‐activated protein kinase pathway (Lu et al. 2002). The second group (*N* = 44) and the third group (*N* = 40) received 0.2 mg/kg of UA (a dose that induces reversible mild PT injury without renal dysfunction) and 4 mg/kg of UA (a dose that induces reversible severe PT injury with significant renal dysfunction) intravenously (Sun et al. 2010), respectively. Rats were anesthetized intraperitoneally with ketamine (75 mg/kg) and xylazine (10 mg/kg) and sacrificed from 18 to 60 h after treatment (*N* = 4 at each time point) for histological examinations and from 18 to 48 h after treatment (*N* = 6 at each time point) for the isolation of tubular cells. Twelve rats without any treatment were used as controls for histological examinations (*N* = 6) and the isolation of tubular cells (*N* = 6).

### Isolation of PT and DT cells

To isolate renal tubular cells and to separate PT cells from DT cells, the method described by Lash et al. was used with slight modifications (Lash et al. 2001). Lash reported that the DT cell population isolated by this method comprised a mixture of cells from the distal convoluted tubules and cortical collecting ducts; cortical and outer medullary thick ascending limb cells were not detected in the PT or DT cell fractions (Lash 1996). Briefly, both kidneys were perfused via the aorta with EGTA‐containing, Ca^2+^‐free HBSS at a flow rate of 8 mL/min for 10 min and with HBSS containing 0.15% (w/v) collagenase (type II) and 2 mM CaCl_2_ for 15 min at a flow rate of 5 mL/min. All buffers were bubbled with 95% O_2_/5% CO_2_ and maintained at 37°C. Isolated renal tubular cells from the cortex and the outer stripe of outer medulla (OSOM) were layered on 35 mL of 45% (vol/vol) isosmotic Percoll solution in 50‐mL polycarbonate centrifuge tubes, which were centrifuged for 30 min at 20,000 × *g* in a Hitachi RPR 20‐2 rotor at 4°C. Cells in the upper quarter and lower quarter of the layer were considered PT cells and DT cells, respectively. Finally, tubular cells were suspended in 2 mL of Krebs–Henseleit buffer and passed through a 32‐*μ*m mesh to remove clumps. The trypan blue exclusion test was used to determine the number of viable cells present in the cell suspensions.

### Cell cycle analysis of isolated tubular cells

#### Evaluation of DNA content using propidium iodide

Freshly isolated PT cells and DT cells in 100 *μ*L of Krebs–Henseleit buffer were permeabilized with Triton X‐100 (final concentration 0.25% v/v) at room temperature for 5 min and then incubated with 50 *μ*g/mL of propidium iodide solution for 15 min. After incubation, the DNA content was measured using an Epics XL flow cytometer (Beckman Coulter, Fullerton, CA). Cells in G0/G1 phase were identified as the population with 2N DNA content, and cells in G2/M phase were identified as the population with 4N DNA content. Cells in S phase were identified as the population having DNA content between that of G0/G1 phase and G2/M phase cells (Darzynkiewicz et al. 1980). For each sample, 10,000 events were analyzed.

#### Separation of G0 phase and G1 phase cells

To separate cells in G1 phase from cells in G0 phase, Hoechst/pyronin Y staining was performed (Crissman et al. 1985). Freshly isolated tubular cells were added to a fixative of ice‐cold 70% ethanol. The cells were fixed for at least 2 h, washed, and suspended at 1 × 10^6^ cells/mL in HBSS. A 1‐mL solution containing 20 *μ*g pyronin Y and 20 *μ*g Hoechst 33342 was added to the cell suspension, and the cells were incubated on ice for 20 min. Cell cycle status was measured using a FACSAria cell sorter (BD Biosciences, San Jose, CA). To determine the RNA content, pyronin Y was excited at 488 nm, and the emission was measured at 562–588 nm. To determine the DNA content, Hoechst 33342 was excited at 355 nm, and the emission was measured at 425–475 nm. Because the RNA content of cells is higher during proliferation than during quiescence (Darzynkiewicz 2010), cells in G0 phase were identified as the population with 2N DNA content and an RNA content lower than that in cells in S and G2/M phases (Crissman et al. 1985; Lemons et al. 2010).

### Immunocytochemistry

To assess the purity of PT cells, immunocytochemistry for megalin, a brush‐border protein in PT cells, was used. Isolated PT cells and DT cells were centrifuged, and each pellet was smeared on a Matsunami adhesive silane‐coated glass slide (Matsunami Glass Ind., Ltd., Osaka, Japan). PT and DT cells were fixed with 2% paraformaldehyde for 10 min and then incubated with 10% donkey serum at room temperature for 60 min. The cells were then exposed to goat anti‐megalin IgG (1:100) with Can Get Signal^®^ solution B at 4°C overnight. The primary antibody was detected with Alexa Fluor 633‐conjugated donkey anti‐goat IgG. For nuclear staining, cells were incubated with DAPI at room temperature for 5 min. The cells were observed with a confocal fluorescence microscope (FV1000; Olympus, Tokyo, Japan).

To discriminate G1 phase cells from G0 phase cells, immunocytochemistry for Cdt1, which is specifically expressed during G1 phase (Wohlschlegel et al. 2000; Nishitani et al. 2001; Xouri et al. 2004, 2007; Sakaue‐Sawano et al. 2008), was also performed. After PT and DT cells were permeabilized with 0.5% Triton X‐100, the cells were incubated with rabbit anti‐Cdt1 IgG (1:100) and then with Alexa Fluor 546‐conjugated goat anti‐rabbit IgG.

### Histological examinations

Both kidneys were dissected after a brief flush with phosphate‐buffered saline. Kidneys were bisected along their longitudinal axis, fixed with 4% paraformaldehyde, and embedded in paraffin. Three‐*μ*m‐thick sections were examined. To evaluate the tubular morphology and the number of tubular cells, the sections were stained with periodic acid‐Schiff.

For immunohistochemistry, kidney sections were deparaffinized, incubated with 5% citrate buffer solution (pH 6.0), and boiled in a microwave for antigen retrieval. The sections were incubated with anti‐Ki67 IgG (1:200) or anti‐vimentin IgG (1:100) primary antibodies at 4°C overnight. The primary antibodies were reacted with Histofine Simple Stain MAX PO or Histofine Simple Stain AP and were visualized using a standard peroxidase‐diaminobenzidine system for Ki67 or Vector Red Alkaline Phosphatase Substrate Kit I for vimentin. Tissue sections were counterstained with hematoxylin. Double immunostaining for Ki67 and vimentin was performed according to a modified method described previously (Fujigaki et al. 2009). As a control, the primary antibody was omitted or replaced with normal serum from the relevant animal. In the control samples, signal was absent or negligible.

Apoptosis was assessed by the terminal uridine nick‐end labeling (TUNEL) technique using ApopTag Plus In Situ Apoptosis Detection Kit (Sun et al. 2010).

For immunofluorescence of p27, kidney sections were incubated with anti‐p27 IgG (1:100) at 4°C overnight. Alexa Fluor 546‐conjugated goat anti‐rabbit IgG was used as a secondary antibody, and nuclei were stained with DAPI. Sections were observed with a BX50 fluorescence microscope (Olympus).

### Western blot of p27

Isolated PT cells were lysed in ice‐cold lysis buffer (300 mM NaCl, 0.3% Triton X‐100, 50 mM Tris‐HCl, pH 7.5) containing protease inhibitors and phosphatase inhibitors. The homogenate was centrifuged at 16,000 × *g* for 15 min at 4°C, and the supernatant was incubated in ImmunoPure Lane Marker Reducing Sample Buffer^®^ with 5% 2‐mercaptoethanol at 99°C for 10 min. A volume containing 15 *μ*g of protein was separated on a sodium dodecyl sulfate‐polyacrylamide gel and transferred to a polyvinylidene difluoride membrane. The membranes were blocked with 5% (wt/vol) skim milk powder in 0.1% Tween‐20 Tris‐buffered saline. Blots were probed with a primary antibody against p27 (1:200) at 4°C overnight. GAPDH (1:10,000) was used as an internal control. The band intensity was quantified using ImageJ software.

### Morphometric analysis

To assess the purity of the isolated PT cells, at least 100 cells per slide were examined, and cells in which polarity of megalin was evident from confocal observation were considered PT cells. Megalin‐negative cells were regarded as DT cells in this study.

The percentage of Cdt1‐positive cells among the isolated PT cells or DT cells was calculated in 10 randomly selected fields on each slide at a magnification of 400×.

For morphometric analysis in the renal sections, PT or DT cells in the cortex and OSOM fields were identified as tubular cells with a PAS‐positive brush‐border, which were also detected on background fluorescence (Vogetseder et al. 2008), or tubular cells without a brush‐border, respectively. PT cells were also differentiated from DT cells as cells with a cuboidal to columnar shape and a centrally located nucleus. The number of PT or DT cells was calculated in 20 randomly selected fields of the cortex and OSOM at a magnification of 400×. The mean number of PT or DT cells per tubule was calculated in 50 cross sections of the PT or DT, respectively, in the cortex and OSOM at a magnification of 400×. The number of Ki67‐positive PT or DT cells and the number of TUNEL‐positive PT cells were counted in 20 randomly selected fields of the cortex and OSOM at a magnification of 400×. The percentage of p27‐positive cells among the PT cells was calculated in 20 randomly selected fields of the cortex and OSOM at a magnification of 400×. The mean score in each rat represented the average number or percentage of PT or DT cells per tubule or field.

### Statistical analysis

All values were expressed as the mean ± SD. Differences between three or more groups were examined for statistical significance by using ANOVA, followed by Tukey's post hoc test. Differences between PT cells and DT cells were assessed using an unpaired *t*‐test (Prism 6; GraphPad Software, San Diego, CA). A *P* value <0.05 was accepted as statistically significant.

## Results

### Isolation of PT cells and DT cells from control rats

**Figure 1.**
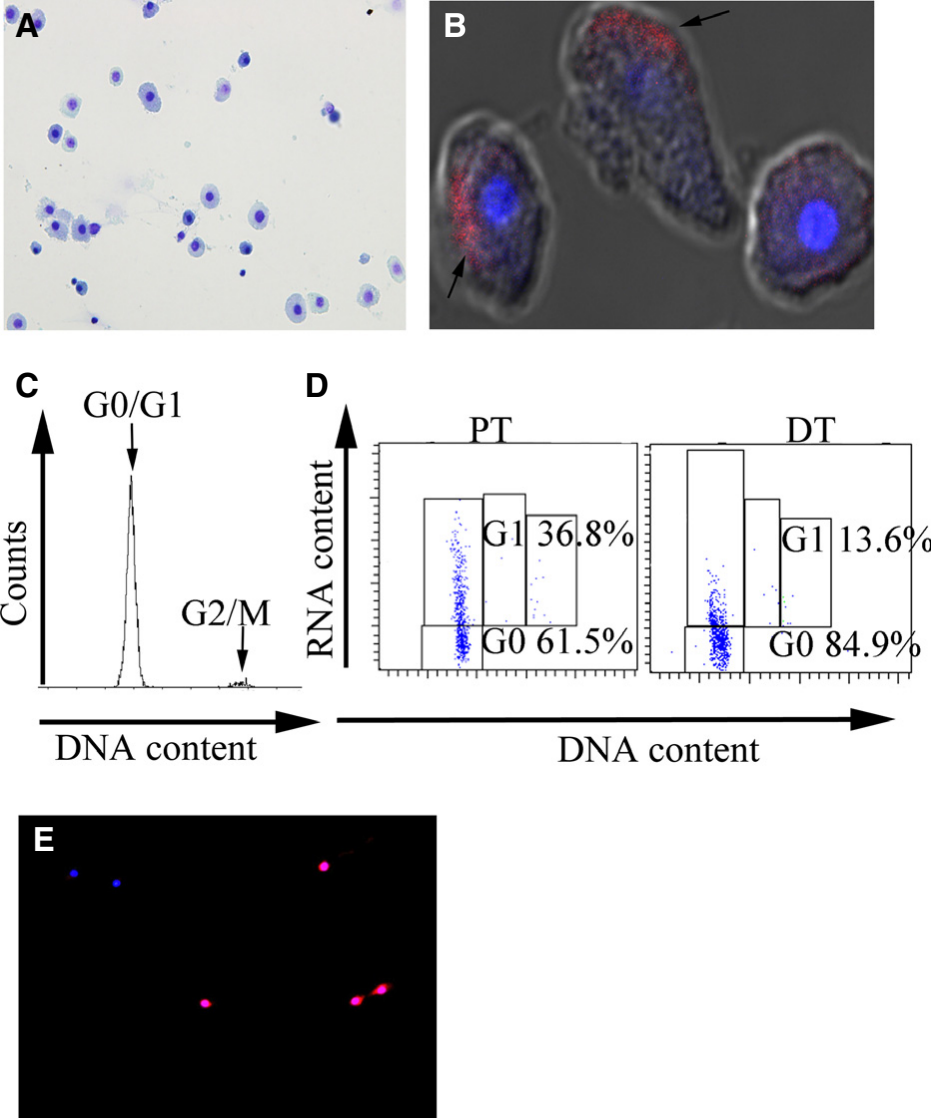
Evaluation of cell cycle status in isolated PT and DT cells. (A) Isolated cells stained with toluidine blue had a cuboidal shape, indicative of tubular cells. Original magnification, 400×. (B) Megalin‐positive tubular cells (red, arrows) in the PT cell fraction observed by confocal immunofluorescent microscopy were identified as PT cells. The nuclei were stained with DAPI (blue). Original magnification, 2000×. (C) Cell cycle analysis of PT cells using propidium iodide and flow cytometry showed that most PT cells were in G0/G1 phase. (D) Cell cycle analysis of PT (left) and DT (right) cells using Hoechst/pyronin Y and flow cytometry showed that the PT cell fraction comprised 61.5% G0 cells and 36.8% G1 cells, while the DT cell fraction comprised 84.9% G0 cells and 13.6% G1 cells. (E) Cdt‐1‐positive cells (red) were identified as G1 phase cells in the PT cell fraction by using immunofluorescent microscopy. The nuclei were stained with DAPI (blue). Original magnification, 1000×.

Most of the isolated cells appeared as single cuboidal cells (Fig. 1A) under an optical microscope, suggesting that the isolated cells were tubular cells. The viability of the cells when evaluated with trypan blue staining was 90.3% ± 3.8% for PT cells and 94.6% ± 4.2% for DT cells. Megalin was positive with polarity in 91.7% ± 3.6% of cells in the PT cell preparation, but in only 7.9% ± 3.7% of cells in the DT cell preparation (Fig. 1B), indicating effective separation of PT and DT cells.

### Cell cycle status in PT and DT cells under physiological conditions

According to flow cytometry analysis of the cellular DNA content, most PT and DT cells were in G0/G1 phase under physiological conditions (Fig. 1C). There were no significant differences in cell cycle status (G0/G1, S, and G2/M phase) between PT and DT cells (Table 1). However, 36.8% ± 5.7% of PT cells were in G1 phase, whereas 13.6% ± 4.4% of DT cells were in G1 phase, when assessed with the Hoechst/pyronin Y method (Fig. 1D), suggesting that the proportion of G1 phase cells was significantly higher among PT cells than among DT cells. The percentage of Cdt1‐positive cells (Fig. 1E) among the isolated PT and DT cells was 30.6% ± 4.5% and 9.9% ± 2.0%, respectively, confirming the results obtained with the Hoechst/pyronin Y method (Fig. 1D).

**Table 1.** Cell cycle status of proximal tubule (PT) and distal tubule (DT) cells under physiological conditions. Data represent the mean ± SD

	G0/G1 (%)	S (%)	G2/M (%)
PT	98.3 ± 0.4	0.4 ± 0.1	1.3 ± 0.3
DT	98.5 ± 0.4	0.3 ± 0.1	1.2 ± 0.3

### Cell cycle status and cell fate in PT and DT cells in response to a proliferative stimulus

**Figure 2.**
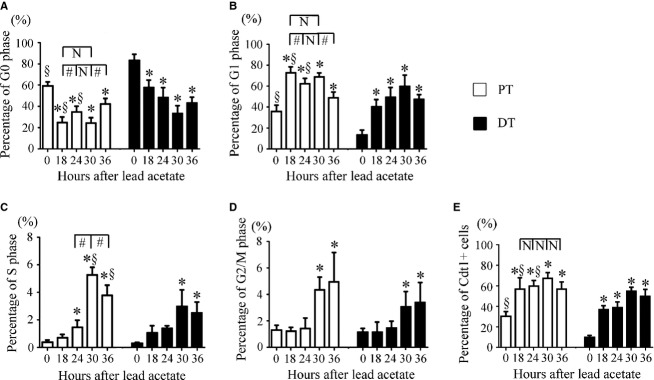
Temporal changes in cell cycle status in the PT and DT after lead acetate administration. The percentages of G0 (A), G1 (B), S (C), and G2/M phase (D) cells and Cdt1‐positive cells (E) in the PT and DT are shown at each time point. Data represent the mean ± SD of six rats. *P < 0.05 versus control rats; ^§^*P *< 0.05 versus DT; ^#^*P *< 0.05. N, no significant difference.

The viability of PT and DT cells isolated from rats administered lead acetate was 87.6% ± 4.2% to 90.2% ± 3.4% and 95.5% ± 2.6% to 97.1% ± 3.7%, respectively. At various times after lead acetate administration, 86.6% ± 4.1% to 92.3% ± 1.5% of the PT cell preparation was megalin‐positive, whereas 10.6% ± 4.3% to 14.8% ± 1.4% of the DT cell preparation was megalin‐positive, indicating effective separation of PT and DT cells. The ratio of G1 to G0 phase cells in the PT and DT increased as early as 18 h after lead acetate administration, peaked at 18–30 h, and decreased at 36 h (Fig. 2A and B). The ratio of G1 to G0 phase cells in the PT was significantly higher than that in the DT from 0 to 24 h. Analysis of G1 phase cells according to Cdt1 expression yielded results similar to those obtained with flow cytometry (Fig. 2E). S phase progression after lead acetate administration started as early as 24 and 30 h in the PT and DT, respectively, and the percentage of S phase cells and G2/M phase cells peaked at 30–36 h in the PT and DT (Fig. 2D and E). The percentages of S phase cells at 30 and 36 h in the PT were significantly higher than in the DT (Fig. 2C), indicating earlier and greater cell cycle progression in the PT relative to that in the DT. The results indicated that the G0‐G1 transition preceded S phase progression in PT and DT cells after a proliferative stimulus.

**Figure 3.**
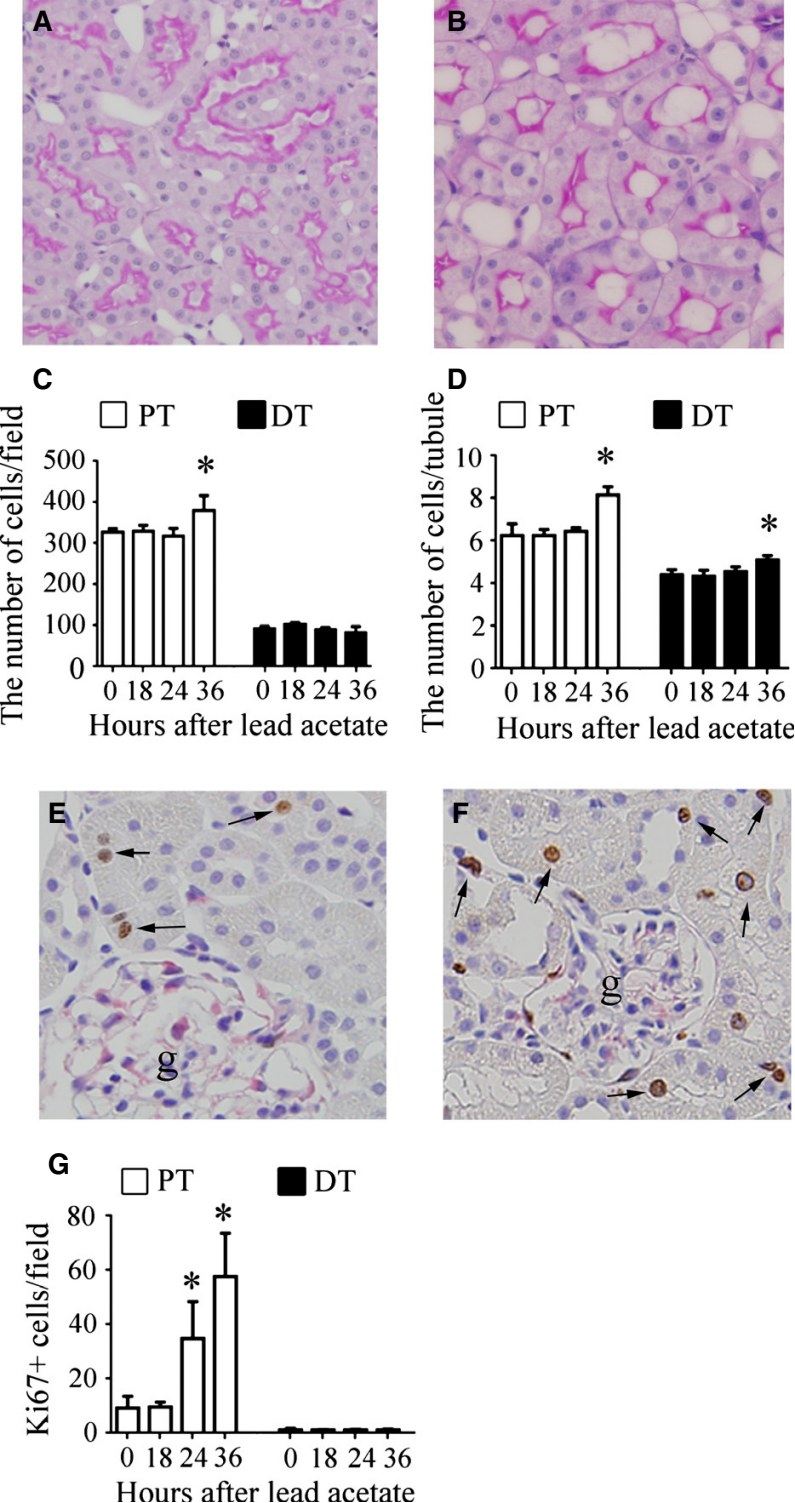
Histological changes in tubular cells after administration of lead acetate. Photomicrographs of periodic acid‐Schiff‐stained renal sections from a control rat (A) and from a rat 36 h after administration of lead acetate, showing hypertrophic changes in PT cells (B). Temporal changes in the number of PT or DT cells per field (C) and in the number of cells per cross section of the PT or DT (D). Data represent the mean ± SD of 4–6 rats. *P < 0.05 versus control rats. Photomicrographs of double‐positive (arrows) immunostaining for Ki67 (brown) and vimentin (red) in a control rat (E) and in a rat 36 h after lead acetate administration (F). Proliferating, nondedifferentiated cells increased after lead acetate administration. g, glomeruli. (G) Temporal changes in the number of Ki67‐positive PT or DT cells after lead acetate administration. Data represent the mean ± SD of 4–6 rats. *P < 0.05 versus control rats.

In histological examinations, an increase in tubule size was apparent 36 h after lead acetate administration (Fig. 3A and B). The number of PT cells increased as early as 36 h after treatment (Fig. 3C). The mean number of cells per cross‐sectional area of tubule increased in the PT and DT as early as 36 h (Fig. 3D). The number of Ki67‐positive proliferating cells increased as early as 24 h in the PT but not in the DT (Fig. 3G).

Few morphologically injured tubular cells were visible in the kidney sections after lead acetate administration (data not shown). Double immunostaining for Ki67 and vimentin at each time point showed that Ki67‐positive proliferating tubular cells did not express vimentin, a phenotypic marker of dedifferentiation (Gröne et al. 1987) (Fig. 3E and F), indicating that proliferating tubular cells stimulated by lead acetate were not dedifferentiated cells but hyperplastic cells, like those observed under physiological conditions (Fujigaki et al. 2009).

### Cell cycle status and cell fate in PT and DT cells in response to a subnephrotoxic stimulus

In rats administered a subnephrotoxic dose of UA, the viability of the isolated PT and DT cells was 86.4% ± 2.1% to 94.1% ± 2.0% and 92.6% ± 2.2% to 98.0% ± 2.8%, respectively. At various time points, 84.0% ± 1.4% to 88.0% ± 5.7% of the PT cell preparation was megalin‐positive, whereas 11.5% ± 3.4% to 13.0% ± 3.5% of the DT cell preparation was megalin‐positive. After a subnephrotoxic dose of UA, the cell cycle status changed only in PT cells.

**Figure 4.**
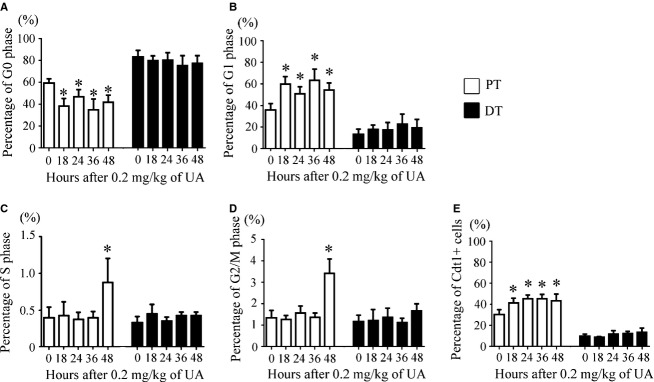
Temporal changes in cell cycle status in the PT and DT after administration of 0.2 mg/kg of UA. The percentages of G0 (A), G1 (B), S (C), and G2/M phase (D) cells and Cdt1‐positive cells (E) in the PT and DT are shown at each time point. Data represent the mean ± SD of six rats. *P < 0.05 versus control rats.

In flow cytometry analysis, the ratio of G1 to G0 phase cells in the PT increased as early as 18 h, and the ratio was sustained until at least 48 h (Fig. 4A and B). Detection of G1 phase cells according to the presence of Cdt1 (Fig. 4E) yielded results similar to those obtained with flow cytometry (Fig. 4B). The percentage of S phase and G2/M phase cells among the PT cells increased at 48 h (Fig. 4C and D).

**Figure 5.**
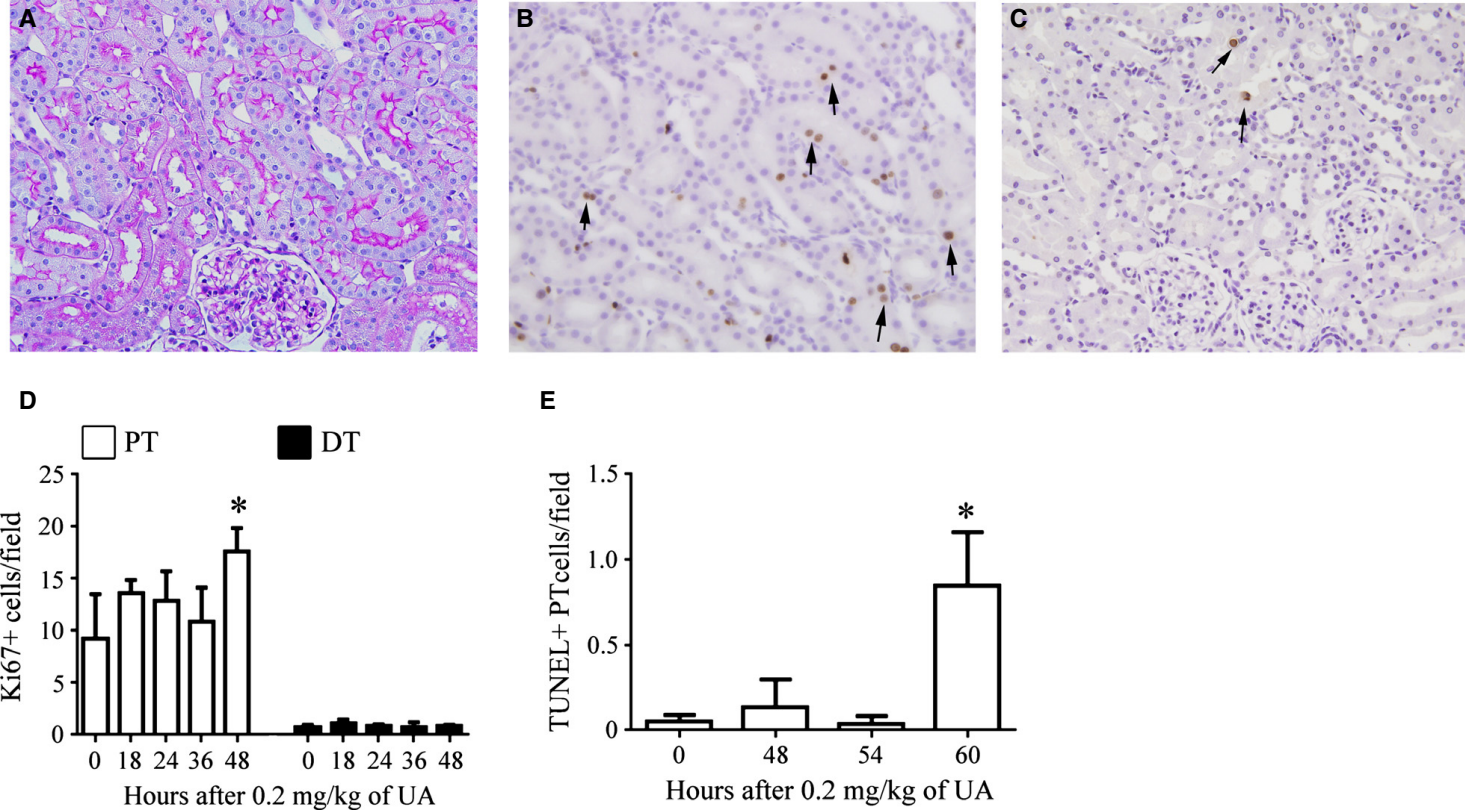
Histological changes in tubular cells after administration of 0.2 mg/kg of UA. (A) Photomicrograph of a periodic acid‐Schiff‐stained renal section, showing that tubular cell structure was almost normal 60 h after UA administration. Original magnification, 400×. (B) Photomicrograph of a Ki‐67‐immunostained (arrows) renal section 48 h after UA administration. Original magnification, 400×. (C) Photomicrograph of a TUNEL‐stained renal section. TUNEL‐positive apoptotic cells (arrows) appeared 60 h after UA administration. Original magnification, 400×. (D) Temporal changes in the number of Ki67‐positive PT or DT cells per field. Data represent the mean ± SD of 4–6 rats. *P < 0.05 versus control rats. (E) Temporal changes in the number of TUNEL‐positive PT cells per field. Data represent the mean ± SD of 4–6 rats. *P < 0.05 versus control rats.

In histological examinations, no tubular cell damage was apparent until 60 h in the PT and DT (Fig. 5A). In the PT, a small number of TUNEL‐positive apoptotic cells were found at this time (Fig. 5C and E). The number of Ki67‐positive cells increased as early as 48 h in the PT, whereas the number in the DT did not increase (Fig. 5B and D). These findings suggested that the G0‐G1 transition preceded cell death and S phase progression after a subnephrotoxic dose of UA.

### Cell cycle status and cell fate in the PT and DT cells in response to a nephrotoxic stimulus

In rats administered a nephrotoxic dose of UA, the viability of PT cells was 81.6% ± 6.3% to 87.8% ± 2.4% until 30 h after UA administration; it then decreased to 40.6% ± 13.5% at 36 h. The percentage of megalin‐positive cells in the PT was 86.2% ± 1.8% to 89.1% ± 3.6% until 30 h after UA administration, but decreased to 69.8% ± 4.6% at 36 h after UA administration. The percentages of megalin‐positive cells and viable cells in the DT cell preparation were 6.8% ± 3.0% to 13.1% ± 3.6% and 91.9% ± 4.1% to 95.4% ± 5.8%, respectively, until 36 h after UA administration. After a nephrotoxic dose of UA, the cell cycle status changed only in PT cells.

**Figure 6.**
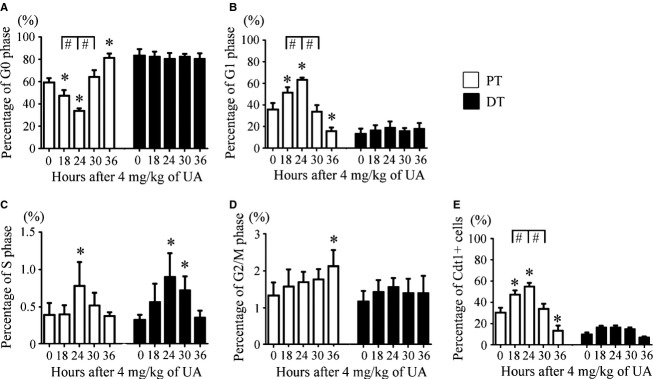
Temporal changes in cell cycle status in PT and DT cells after administration of 4 mg/kg of UA. The percentages of G0 (A), G1 (B), S (C), and G2/M (D) phase cells and Cdt1‐positive cells (E) in the PT and DT are shown at each time point. Data represent the mean ± SD of six rats. *P < 0.05 versus control rats; ^#^*P *< 0.05.

The ratio of G1 to G0 phase cells in the PT increased as early as 18 h, peaked at 24 h, and decreased from 30 h (Fig. 6A and B). Detection of G1 phase cells according to the presence of Cdt1 (Fig 6E) yielded results similar to those obtained with flow cytometry (Fig. 6B). The percentages of S phase and G2/M phase cells among the PT cells transiently increased at 24 and 36 h, respectively (Fig. 6C and D). High cell viability and effective separation of PT and DT cells were observed until 30 h, but the percentages of viable PT cells and megalin‐positive PT cells decreased to approximately 40% and 70%, respectively, at 36 h, suggesting that the findings for cells isolated at 36 h were not reliable.

**Figure 7.**
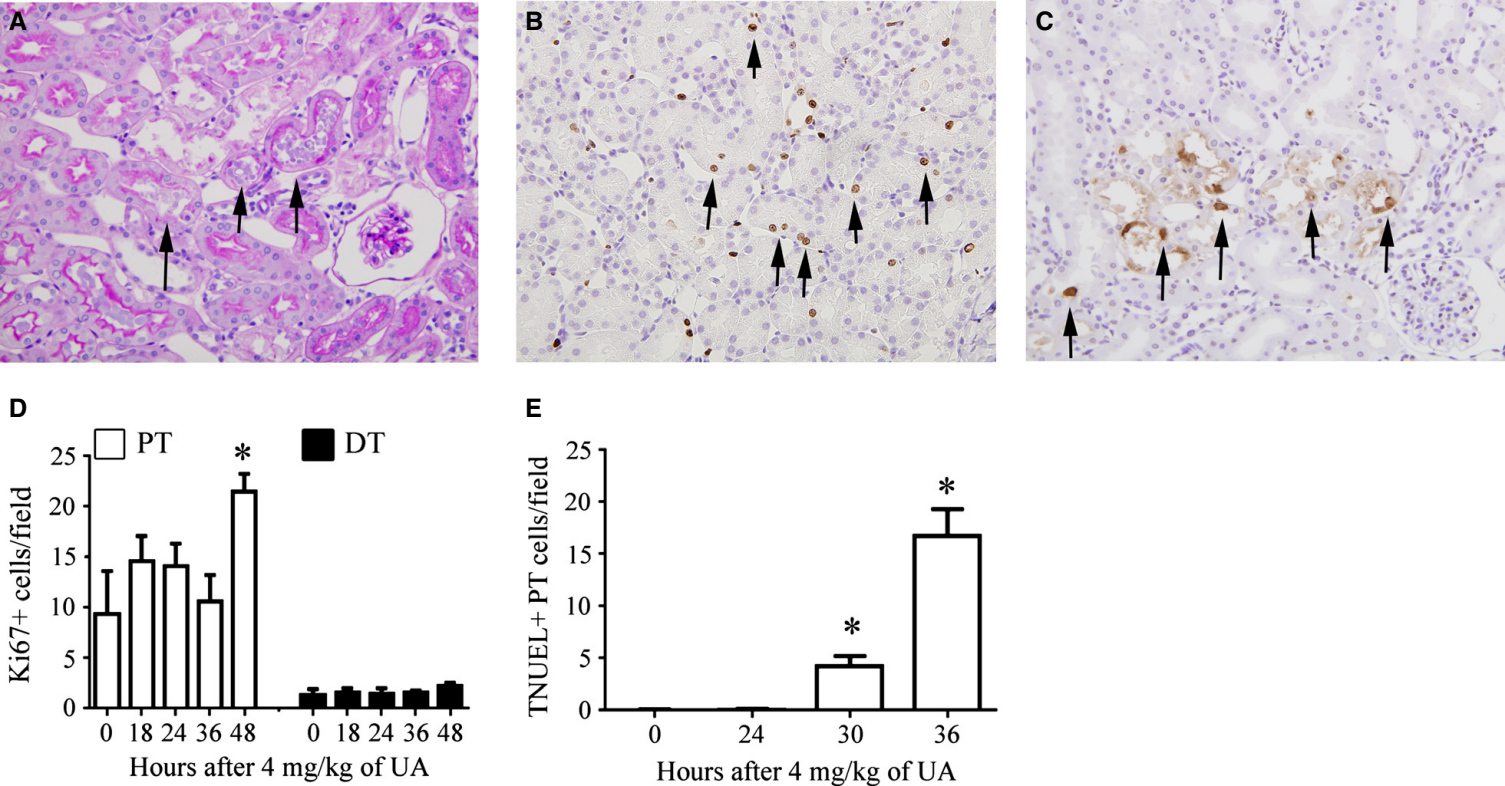
Histological changes in tubular cells after administration of 4 mg/kg of UA. (A) Photomicrograph of a periodic acid‐Schiff‐stained renal section, showing detached PT cells in the PT lumen (arrows) at the corticomedullary junction 30 h after UA administration. Original magnification, 400×. (B) Photomicrograph of a Ki‐67‐immunostained renal section 48 h after UA administration. Original magnification, 400×. (C) Photomicrograph of a TUNEL‐stained renal section. TUNEL‐positive apoptotic cells (arrows) in the corticomedullary junction appeared 30 h after UA administration. Original magnification, 400×. (D) Temporal changes in the number of Ki67‐positive PT or DT cells per field. Data represent the mean ± SD of 4–6 rats. *P < 0.05 versus control rats. (E) Temporal changes in the number of TUNEL‐positive PT cells per field. Data represent the mean ± SD of 4–6 rats. *P < 0.05 versus control rats.

In histological analyses, necrotic cells or cells detached from the tubular basement membrane were found in the PT as early as 30 h (Fig. 7A), and TUNEL‐positive apoptotic cells were observed at the same time (Fig. 7C and E). The number of Ki67‐positive cells increased as early as 48 h in the PT but not after the first 48 h in the DT (Fig. 7B and D). The findings suggested that a nephrotoxic dose of UA promoted the G0‐G1 transition and the accumulation of G1 phase cells before cell death and S phase progression in the PT. They also suggested that S phase progression in PT cells started more rapidly in rats administered a nephrotoxic dose of UA than in rats administered a subnephrotoxic dose of UA.

### p27 expression in PT cells in response to a proliferative or injurious stimulus

**Figure 8.**
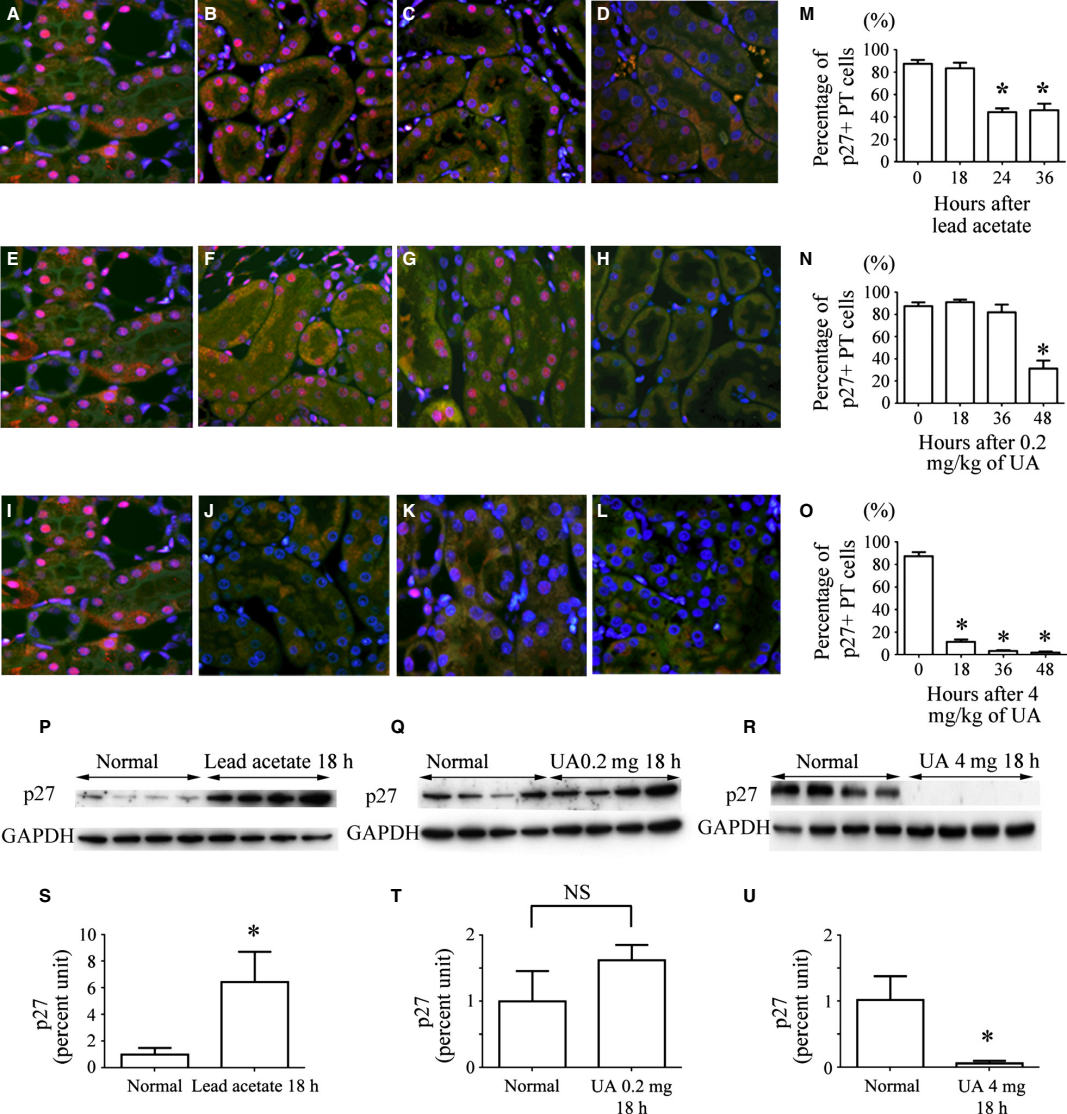
p27 in the PT. Photomicrographs of p27 (red) immunofluorescence in renal sections from control rats (A, E, I), rats treated with lead acetate (B, 18 h; C, 24 h; D, 36 h), rats treated with 0.2 mg/kg of UA (F, 18 h; G, 36 h; H, 48 h), and rats treated with 4 mg/kg of UA (J, 18 h; K, 36 h; L, 48 h). Nuclei were stained with DAPI (blue). (M–O) Morphometric analysis of temporal changes in the percentage of p27‐positive PT cells in rats treated with lead acetate (M), rats treated with 0.2 mg/kg of UA (N), and rats treated with 4 mg/kg of UA (O). Data represent the mean ± SD of 4–6 rats. *P < 0.05 versus control rats. (P–R) Western blot with antibodies for p27 and GAPDH of protein isolated from the PT cells of control rats, rats treated with lead acetate, rats treated with 0.2 mg/kg of UA, and rats treated with 4 mg/kg of UA. (S–U) p27 levels relative to GAPDH levels were quantified by densitometry. Data represent the mean ± SD of four rats. *P < 0.05 versus control rats.

In immunofluorescence analysis, 87.4% ± 3.0% of PT cells expressed p27 in the nucleus under control conditions (Fig. 8A, E and I). In rats treated with lead acetate, the percentage of p27‐positive cells in the PT decreased as early as 24 h (Fig. 8B–D and M). In rats administered a subnephrotoxic dose of UA, the percentage of p27‐positive cells in the PT decreased as early as 48 h (Fig. 8F–H and N). In rats administered a nephrotoxic dose of UA, the percentage of p27‐positive cells in the PT decreased drastically as early as 18 h (Fig. 8J–L and O).

Because p27 immunofluorescence might not exactly reflect p27 protein expression, p27 protein levels were examined with western blot 18 h after a proliferative or injurious stimulus, when acceleration of the G0‐G1 transition and accumulation of G1 phase cells were observed before S phase progression. The level of p27 increased markedly 18 h after lead acetate administration (Fig. 8P and S). The p27 level did not significantly change 18 h after the administration of a subnephrotoxic dose of UA (Fig. 8Q and T). In contrast, almost no p27 protein was detected 18 h after the administration of a nephrotoxic dose of UA (Fig. 8R and U).

## Discussion

Using flow cytometry, we found that most PT and DT cells from control rats were in the G0/G1 phase, and that the ratio of G1 to G0 phase cells was high. We also found that the PT had a higher percentage of G1 phase cells than did the DT. The high G1 to G0 ratio in PT cells is consistent with the findings of Vogetseder et al. (2008), who immunostained for cyclin D1 under physiological conditions. Metabolic activity is lower in G0 phase cells than in cycling cells, including G1 phase cells (Choder 1991; Fuge et al. 1994). Thus, the high proportion of G1 phase cells might contribute to the metabolic activity in the PT. It is also possible that many PT cells resting in G1 phase can rapidly enter the cell cycle and divide when needed.

In this study, we tested whether G1 phase cells in the PT progressed promptly to S phase after a proliferative or injurious stimulus. In response to a proliferative stimulus, the ratio of G1 to G0 phase cells increased before S phase progression in the PT and DT, with no tubular cell injury apparent in renal tissues. Administration of subnephrotoxic/nephrotoxic doses of UA also increased the ratio of G1 to G0 phase cells in the PT before the appearance of TUNEL‐positive apoptotic cells in renal tissues. Ki67‐positive cells did not express vimentin after lead acetate administration, indicating that proliferating cells were hyperplastic without phenotypic changes. Our previous study showed that bromodeoxyuridine‐positive cells were positive for vimentin after UA administration, indicating that the proliferating cells acquired a dedifferentiated phenotype (Fujigaki et al. 2006, 2009). The findings suggest that, after proliferative or injurious stimuli, cells in the PT first commit to the G0‐G1 transition; existing G1 phase cells arrest and accumulate instead of progressing to S phase in both types of PT regeneration (with or without phenotypic change). Cells check for and repair DNA damage in G1 phase before S phase progression. Thus, G1 phase cells already present in the PT may arrest in order to do so.

In rats administered a nephrotoxic dose of UA, the ratio of G1 to G0 phase cells in the PT increased initially, but decreased 30 h after UA administration. There are a couple explanations for this phenomenon. First, in response to DNA damage, proteolysis of Cdt1, an essential protein for chromosome replication that is specifically expressed in G1 phase cells (Wohlschlegel et al. 2000; Nishitani et al. 2001; Xouri et al. 2004, 2007; Sakaue‐Sawano et al. 2008), has been demonstrated in a number of organisms (Hall et al. 1988; Higa et al. 2003; Ralph et al. 2006; Cook 2009). Upon proteolysis of Cdt1, some cells in G1 phase transitioned to G0 phase (Xouri et al. 2004). It has been reported that apoptosis can occur at G1 phase (Diez‐Roux et al. 1999) and that G0 phase cells are protected from some toxic insults or oxidative stress (Naderi et al. 2003; Allen et al. 2006; Legesse‐Miller et al. 2012). Because only a small number of TUNEL‐positive cells were present in renal tissues at 30 h, the decreased number of G1 phase cells in the PT could reflect an active G1‐G0 transition process. Second, a large number of PT cells die after a nephrotoxic dose of UA (Fujigaki et al. 2006). In this study, the viability of isolated PT cells was 40% at 36 h. The low viability of the isolated PT cells at 36 h indicates that some dead cells were included in the isolated PT cells, in which some type of protein and RNA related to cell cycle should be degraded, influencing the cell cycle status. Thus, we conclude that the findings regarding the cells isolated at 36 h were not reliable. If many isolated PT cells are already dysfunctional or detached at 30 h, they might not express features characteristic of G1 phase cells.

In contrast to PT cells, the cell cycle status and viability in the DT cells did not change in response to the subnephrotoxic/nephrotoxic dose of UA. Generally, PT cells have been shown to be more vulnerable to nephrotoxic agents than DT cells, probably because PT cells are more exposed to the agents than DT cells owing to their absorption mechanism for such agents. Based on our observation, DT cells had a higher percentage of cells in the G0 phase than PT cells. Thus, whether cells in the G0 phase have resistance to nephrotoxic stimuli in DT cells remains unclear.

We examined the expression of p27, a cyclin‐dependent kinase inhibitor thought to be a G1 phase mediator. Two main pathways for p27 degradation have been reported. The first pathway is mediated by the Kip1 ubiquitination‐promoting complex ubiquitin ligase and is responsible for the degradation of p27 at the G0‐G1 transition (Kamura et al. 2004). The second pathway is mediated by the S phase kinase‐associated protein (SKP) 2‐dependent SKP‐Cullin‐F‐box E3 ligase, which promotes the polyubiquitylation and subsequent proteasomal degradation of p27 at the G1‐S transition (Sutterlüty et al. 1999). The results of p27 immunofluorescence in renal tissues indicated that the percentage of p27‐positive PT cells decreased before or at the time when the percentage of S phase cells increased in response to proliferative and injurious stimuli. This suggests that the reduced expression of p27 is associated with promotion of the G1‐S transition.

The number of p27‐positive cells was not changed 18 h after the administration of lead acetate or a subnephrotoxic dose of UA, when the G0‐G1 transition and G1 arrest were enhanced. Because protein levels are difficult to quantify with immunofluorescence staining, we used western blot to assess p27 levels in isolated PT cells 18 h after the stimulus. p27 protein was increased in isolated PT cells 18 h after the proliferative stimulus. Since most of the PT cells expressed p27 under a controlled condition, we thought it might be difficult to accurately assess the increased expression level of p27 in PT after the stimulus by using immunofluorescence analysis. p27 levels in a cell should decrease at the G0‐G1 transition, but increase with G1‐arrest. Thus, the increase in p27 in the PT cell population may reflect the increased p27 protein level in cells arrested in G1 phase. p27 protein levels in the PT cells did not change 18 h after the administration of the subnephrotoxic dose of UA, suggesting that the reduction in p27 protein associated with the G0‐G1 transition was almost balanced by the increase in p27 protein level associated with G1 arrest. The findings suggest that p27 protein is involved in the G0‐G1 transition and in G1 arrest in cells exposed to a proliferative or subnephrotoxic stimulus. In contrast, almost no p27 protein was detected in PT cells 18 h after the administration of a nephrotoxic dose of UA, consistent with the p27 immunofluorescence results. At this stage, the G0‐G1 transition was promoted in PT cells, and at 24 h, the percentage of S phase cells was slightly and transiently increased. During S phase progression, p27 should be degraded. In response to a nephrotoxic dose of UA, it is possible that PT cells begin to enter cell cycle rapidly as p27 is degraded, with most G0 phase cells transitioning first to G1 phase and then progressing to S phase. However, with the G1‐S transition, the number of cells in S phase does not increase continuously because most PT cells die or become dysfunctional as a result of the nephrotoxic insult; only a small number of PT cells subsequently proliferate to repair the damaged PT (Sakakima et al. 2008). Because the p27 status was not elucidated in this study, further studies are needed to clarify which p27 phosphorylation status is important for maintaining cells in the G1 phase in response to proliferative or injurious stimuli.

It is important to note the methodological limitations of this study. First, because we mainly focused on PT cells in this study, DT cells were defined only based on a paucity of megalin staining on cytological analysis. Thus, we cannot rule out the presence of epithelial cells that are not from PT or DT in DT fraction. Second, additional information on co‐staining megalin with Ki67, TUNEL, and p27 would be more reliable to identify PT cells in renal tissues. Unfortunately, because the anti‐megalin antibody used in this study did not work well in the renal specimen fixed with paraformaldehyde, we differentiated PT cells morphologically from DT cells. Another reason was that according to our previous observation, megalin began to disappear because of the phenotypic change in the PT cells as early as 72 or 48 h after exposure to the subnephrotoxic or nephrotoxic dose of UA, respectively (Fujigaki et al. 2009).

In summary, we showed that most PT and DT cells from control rats were in G0/G1 phase and that the ratio of G1 to G0 phase cells was high. The ratio of G1 to G0 phase cells was higher in PT cells than in DT cells. After a proliferative or injurious stimulus, a prompt G1‐S transition was not observed. Rather, G0‐G1 transition and G1 arrest preceded S phase progression in PT cells. This suggests that the rapid generation of G1 phase cells and the repair of DNA damage in existing G1 phase cells occur at the same time in the PT, before S phase progression, when p27 should be involved. Taken together, the high G1 to G0 cell ratio among PT cells and the rapid accumulation of G1 phase cells may be a preparatory state that facilitates safe, timely, and explosive cell proliferation in response to acute PT damage.

## Acknowledgements

The authors express their gratitude to Kiyoshi Shibata (Equipment Center, Hamamatsu University School of Medicine, Hamamatsu, Japan) for his valuable assistance with technical support.

## Conflict of Interest

We declare that we have no conflict of interest.
